# Assessment of the Field Utility of a Rapid Point-of-Care Test for SARS-CoV-2 Antibodies in a Household Cohort

**DOI:** 10.4269/ajtmh.21-0592

**Published:** 2021-11-24

**Authors:** Mehal Churiwal, Kelly D. Lin, Salman Khan, Srijana Chhetri, Meredith S. Muller, Kathleen Tompkins, Judy Smith, Christy Litel, Maureen Whittelsey, Christopher Basham, Tyler Rapp, Carla Cerami, Lakshmanane Premkumar, Jessica T. Lin

**Affiliations:** ^1^ Institute of Global Health and Infectious Diseases, University of North Carolina School of Medicine, Chapel Hill, North Carolina;; ^2^ Department of Microbiology and Immunology, University of North Carolina School of Medicine, Chapel Hill, North Carolina;; ^3^ MRC Unit The Gambia at the London School of Hygiene & Tropical Medicine, Fajara, The Gambia

## Abstract

Point-of-care (POC) tests to detect SARS-CoV-2 antibodies offer quick assessment of serostatus after natural infection or vaccination. We compared the field performance of the BioMedomics COVID-19 IgM/IgG Rapid Antibody Test against an ELISA in 303 participants enrolled in a SARS-CoV-2 household cohort study. The rapid antibody test was easily implemented with consistent interpretation across 14 users in a variety of field settings. Compared with ELISA, detection of seroconversion lagged by 5 to 10 days. However, it retained a sensitivity of 90% (160/177, 95% confidence interval [CI] 85–94%) and specificity of 100% (43/43, 95% CI 92–100%) for those tested 3 to 5 weeks after symptom onset. Sensitivity was diminished among those with asymptomatic infection (74% [14/19], 95% CI 49–91%) and early in infection (45% [29/64], 95% CI 33–58%). When used appropriately, rapid antibody tests offer a convenient way to detect symptomatic infections during convalescence.

Point-of-care (POC) tests to detect SARS-CoV-2 antibodies have great potential to expand global capacity for COVID-19 testing and surveillance.^1^^–^^4^ As their use extends beyond traditional clinical settings to help determine serostatus after infection or vaccination, evaluating test performance against laboratory assays at the POC—not just in controlled laboratory settings^5^^–^^7^—is essential. The BioMedomics COVID-19 IgG/IgM Rapid Antibody Test is a lateral flow immunoassay (LFA) that detects antibodies to the SARS-CoV-2 spike protein receptor binding domain (RBD). Like most LFA antibody tests, it requires only a few drops of finger-prick blood to yield results within 10 to 15 minutes (Figure 1).^8^ Although the assay was removed from the U.S. Food and Drug Administration list of rapid antibody tests authorized for emergency use and distribution in the United States,^9^ the BioMedomics LFA was validated by two independent sample repositories,^4^^,^^5^ has obtained CE certification in the European Union, and is being used globally in hospitals, clinics, and government agencies.

**Figure 1.**
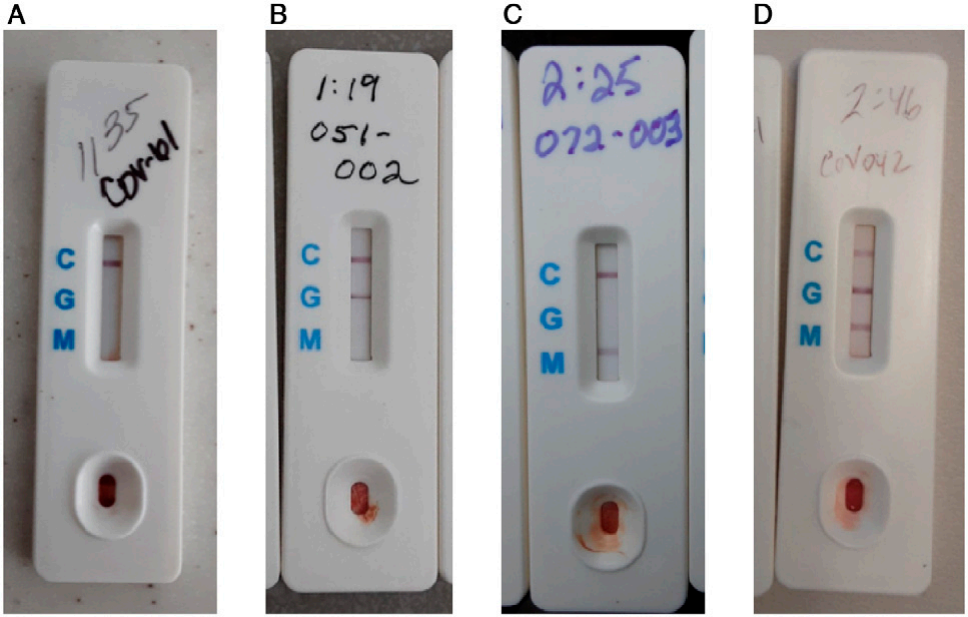
BioMedomics LFA test results for (**A**) negative reading, (**B**) only IgG positive reading, (**C**) only IgM positive reading, and (**D**) both IgG and IgM positive readings. This figure appears in color at www.ajtmh.org.

Here, we describe its field use and performance characteristics compared with a laboratory ELISA among 303 participants enrolled in the University of North Carolina at Chapel Hill (UNC) COVID-19 Household Transmission (CO-HOST) study (NCT04445233).^10^ The CO-HOST study received ethical approval from the Institutional Review Board at UNC. It was conducted in central North Carolina between April and November 2020, before the availability of COVID-19 vaccination (Supplemental Figure 1).^10^ Adults were enrolled 1 to 5 days after testing positive for SARS-CoV-2 by polymerase chain reaction (PCR) at the UNC Respiratory Diagnostic Center (RDC), if they lived with one or more household contacts who also agreed to participate in the study.

The LFA was widely accepted and easily implemented in different settings. BioMedomics rapid antibody tests were performed at the POC on 303 participants at study enrollment (D1) and/or roughly 4 weeks later (∼D28), totaling 573 BioMedomics LFA tests collected throughout the study (Supplemental Figure 2). Testing was performed in various locations, including a mobile unit van (*n* = 359), participants’ homes (*n* = 99), and the RDC (*n* = 86). Ninety-nine percent (303/306) of those offered the rapid antibody test consented to testing; the three participants who declined were < 18 years of age. In comparison, venipuncture for serologic testing was declined or unsuccessful in 10% (30/299) of those eligible for collection. (Venipuncture was not attempted in four participants who were either < 6 years old or admitted to the hospital.)

LFA test results demonstrated good inter-user congruence in terms of overall positive versus negative status. Tests were read and entered into a REDCap study database via tablet by 14 study staff over the course of the study, either by the staff member performing the finger prick (e.g., study nurse, phlebotomist) or by an undergraduate research assistant. Results were identified as positive or negative for IgM and IgG, with positive bands further specified as strong (definitively present at a glance) or faint (present, but may be difficult to see at a glance) (Figure 1). An LFA test was considered positive if either or both the IgM and IgG bands were at least faintly present. Overall, 94% (239/256) of positive BioMedomics LFAs displayed an IgG band (either IgM−/IgG+ or IgM+/IgG+), with 64% (165/256) showing a strong IgG band. A blind reader had 97% agreement (305/313) with the field results on overall test positivity (IgM+ or IgG+) through independent assessment of LFA tests that were captured in photographs (313/573). The majority of discrepant tests (5/8) were read as negative in the field but positive with only a faint IgM band in the blind read.

Serum samples collected alongside the BioMedomics LFA were tested using an in-house ELISA based on the SARS-CoV-2 spike RBD. This anti-RBD ELISA was previously evaluated on a large panel of well-characterized samples and shown to have high sensitivity (98%) and specificity (100%) for detecting SARS-CoV-2 infection 9 days after symptom onset.^11^ On the basis of this previous testing, an ELISA test was considered positive if the total Ig optical density (OD) was at least 0.376. The levels of RBD binding antibodies correlated highly with neutralizing antibody titers. Using 20 µL of serum spiked with human anti-RBD monoclonal IgG (CR3022), the detection limit of the anti-RBD ELISA is 60 pg of CR3022. In comparison, the analytic sensitivity of the BioMedomics LFA was found to be 20 and 10 ng for strong and faint bands, respectively (Supplemental Figure 3). On the basis of two titrations performed, 20 ng of CR3022 corresponds to approximately 1.7 OD in the ELISA.

Despite the lower analytic sensitivity of the LFA compared with ELISA, the BioMedomics rapid test showed excellent sensitivity and specificity at 1-month convalescent testing compared with ELISA as the gold standard. On the basis of 220 tests administered on or around D28, of which 177 were from individuals who tested ELISA-positive at D28 and 43 were from individuals who tested ELISA-negative at D28, the sensitivity of the LFA (any positive bands) was 90% (160/177, 95% confidence interval [CI]: 85–94%) and specificity was 100% (43/43, 95% CI: 92–100%) (Supplemental Figure 2). This translates to a positive predictive value of 100% and negative predictive value of 72% in this highly exposed outpatient cohort. In keeping with excellent sensitivity at D28, the LFA detected 91% (51/56) of those who seroconverted over the course of the study based on ELISA testing (ELISA-negative at D1 then ELISA-positive at D28).

For ELISA-positive cases that were missed by LFA at D28 (*n* = 17), the ELISA IgG OD was generally lower than the measured analytic sensitivity of the BioMedomics LFA (median OD: 0.7, interquartile range: 0.3–1.6). Among these 17 low-titer participants, nine had confirmatory positive PCR results, three were asymptomatic, and the remaining five each reported less than 1 week of symptoms.

At D1, when most infected participants were early in infection and presumably just starting to generate antibodies, the LFA showed decreased sensitivity compared with ELISA (LFA-ELISA discrepant results at D1 are depicted as hollow and gray circles in Figure 2; these were interpreted regardless of subsequent results at D28). In 279 tests completed at D1, of which 64 were from ELISA antibody-positive individuals and 124 from ELISA antibody-negative individuals, the LFA was positive in only 45% (29/64, 95% CI: 33–58%) of ELISA-positive samples. As expected, samples that were LFA-negative displayed a lower ELISA total Ig OD compared with samples that were both ELISA and LFA-positive (median OD 0.6 versus 2.0 for ELISA+/LFA– versus ELISA+/LFA+ samples, respectively, *P* < 0.0001) (Supplemental Figure 4). There were 7 D1 samples that were ELISA-negative, yet LFA-positive (Supplemental Figure 2). Five of seven were confirmed as SARS-CoV-2-positive based on PCR or subsequent D28 ELISA antibody testing. Both of the remaining two were IgM+/IgG– and most likely false positives.

**Figure 2.**
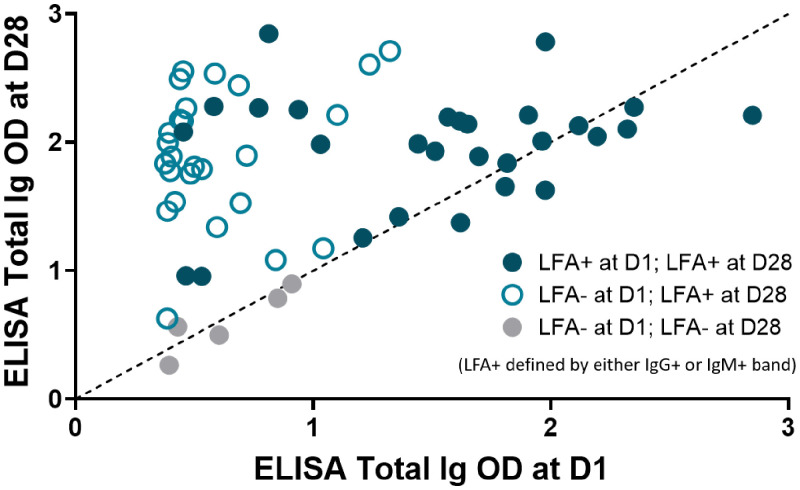
ELISA Total Ig OD at Day 1 of study enrollment (D1) and Day 28 (D28) in individuals who were already ELISA-positive at D1. Data are categorized based on positivity of the BioMedomics LFA at D1 vs D28. The threshold of ELISA positivity was 0.376 OD. ELISA Ig titers increased from D1 to D28, with most LFA-ELISA discrepancies at D1 occurring among those with lower Ig titers at D1. This figure appears in color at www.ajtmh.org.

Sensitivity of the LFA was also diminished among those with asymptomatic SARS-CoV-2 infection. As part of the study, nasopharyngeal and/or nasal mid-turbinate swabs were collected at enrollment and on D7, D14, and D21. These were tested using a CDC reverse transcriptase quantitative PCR protocol authorized for emergency use.^12^ We defined asymptomatic cases as those who reported no or one cumulative symptoms in daily symptom diaries collected over 14 days, as long as the one symptom was not fever, shortness of breath, or anosmia. Among those who were SARS-CoV-2 PCR-positive but asymptomatic, the sensitivity of the LFA at D28 was 74% (14/19, 95% CI: 49–91%), compared with 92% (11/12, 95% CI: 62–100%) for the ELISA (Figure 3).

**Figure 3.**
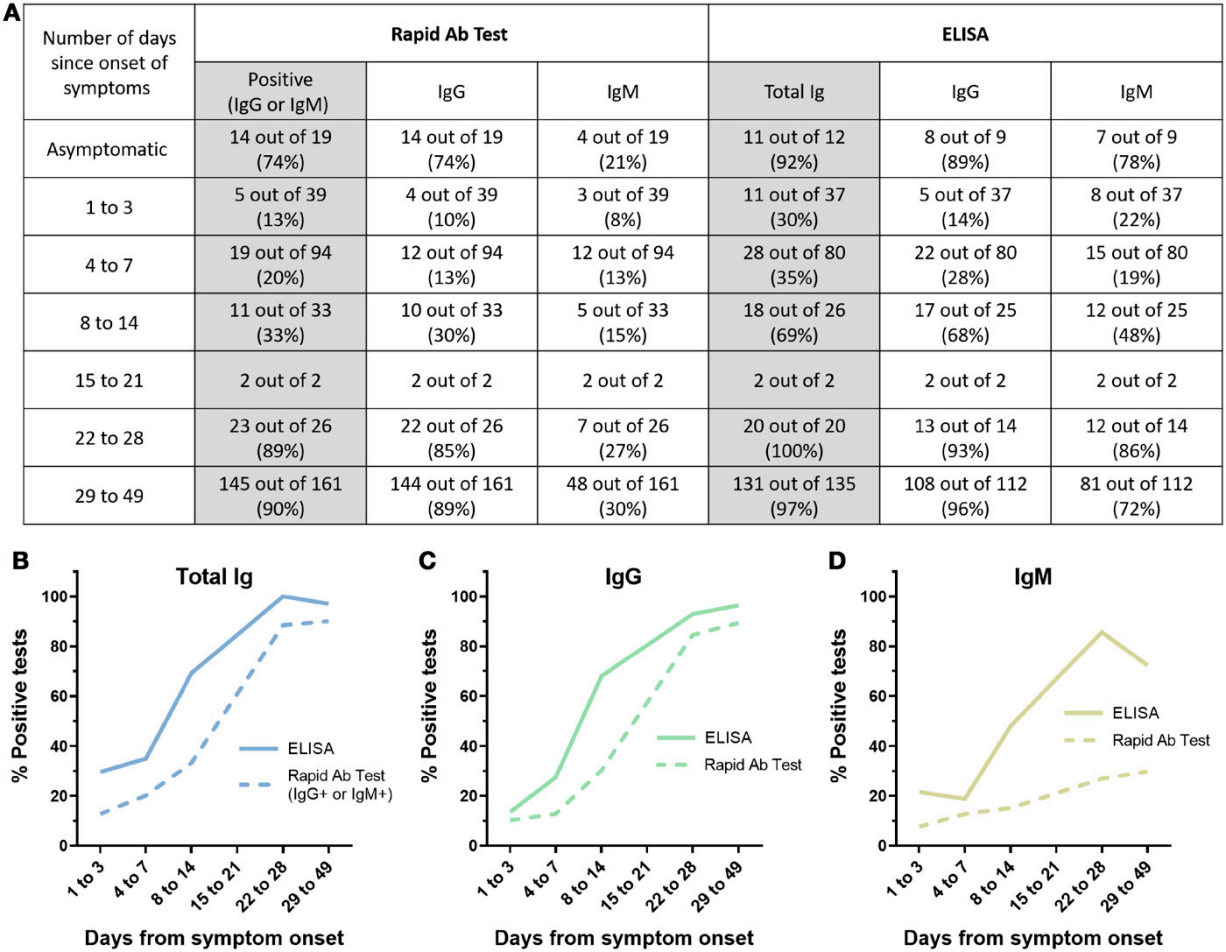
Time to antibody positivity. (**A**) Using samples collected from COVID+ participants, sensitivity was calculated based on the number of days from symptom onset on the day of sample collection. Data from asymptomatic participants is based on samples collected at D28 only. Excluding data points for the interval of 15 to 21 days, graphs were made for (**B**) ELISA total Ig and BioMedomics LFA IgG/IgM, (**C**) ELISA IgG and BioMedomics LFA IgG, and (**D**) ELISA IgM and BioMedomics LFA IgM. This figure appears in color at www.ajtmh.org.

Finally, we compared time to positivity for the BioMedomics LFA versus ELISA. Compared with ELISA, the LFA lagged in detecting seroconversion by 5 to 10 days (Figure 3B). To look at time to antibody positivity over time, we selected all SARS-CoV-2-positive individuals in the study, defined as those who were PCR-positive at any study visit or ELISA-positive at D28. Participants had variable symptom onset ranging anywhere from 2 weeks before study D1 to 2 weeks after D1. This enabled us to assess the proportion of individuals who tested positive by each test according to the number of days since symptom onset (Figure 3A). ELISA testing was positive in a majority of participants roughly 1 week after symptom onset, whereas seroconversion occurred in the majority of cases 2 to 3 weeks after symptom onset. IgM testing was less likely to be positive in either assay.

Widespread deployment of POC testing for SARS-CoV-2 could transform the global COVID-19 response, helping individuals quickly understand whether they were previously infected or developed a robust response to vaccination, and informing health officials on the level of community protection that may be present. Antibodies to the spike protein RBD, measured by the BioMedomics LFA and by many similar rapid antibody tests, correlate with neutralizing antibody titers^11^^,^^13^ and account for ∼90% of the neutralizing activity in SARS-CoV-2 immune sera.^14^^,^^15^ However, threshold levels of neutralizing antibodies for SARS-CoV-2 immunity and protection have not been ascertained because even low levels of neutralizing antibodies have been shown to provide protection in nonhuman primates.^16^ Our study provides insight into how SARS-CoV-2 lateral flow assays are likely to perform in POC settings when used by individuals other than laboratory professionals.

Reassuringly, performance of the BioMedomics COVID-19 IgG/IgM Rapid Antibody Test at the POC was congruent with its analytic sensitivity and matched previous laboratory assessments of its performance.^17^^,^^18^ When used ∼1 month after the onset of symptoms, it demonstrated robust sensitivity (90%) and complete specificity (100%) in a heavily exposed household cohort. Sensitivity was diminished early in infection and in those who never developed symptoms (74%), likely due to the presence of lower antibody titers.^19^ The number of true false positives early in infection, when serologic testing is not recommended, appeared low, with only two detected among 499 tests, due to false-positive IgM bands. Additional POC studies should be completed in lower prevalence cohorts, in those 6 to 12 months postrecovery, and vaccinated individuals to add to our understanding of how SARS-CoV-2 rapid antibody tests will perform in real-world settings where they are most needed.

## Supplemental tables


Supplemental materials

## References

[1] Boum Y 2021. Performance and operational feasibility of antigen and antibody rapid diagnostic tests for COVID-19 in symptomatic and asymptomatic patients in Cameroon: a clinical, prospective, diagnostic accuracy study. Lancet Infect Dis 21: 1089–1096. 33773618 10.1016/S1473-3099(21)00132-8 PMC7993929

[2] Iruzubieta P 2021. Feasibility of large-scale population testing for SARS-CoV-2 detection by self-testing at home. Sci Rep 11: 9819. 33972607 10.1038/s41598-021-89236-x PMC8110575

[3] Peeling RW Olliaro P, 2021. Rolling out COVID-19 antigen rapid diagnostic tests: the time is now. Lancet Infect Dis 21: 1052–1053. 33773617 10.1016/S1473-3099(21)00152-3 PMC7993925

[4] Augustine R 2020. Rapid antibody-based COVID-19 mass surveillance: relevance, challenges, and prospects in a pandemic and post-pandemic world. J Clin Med Res 9: 3372. 10.3390/jcm9103372 PMC7589650 33096742

[5] Conklin SE 2021. Evaluation of serological SARS-CoV-2 lateral flow assays for rapid point-of-care testing. J Clin Microbiol 59: e02020–20. 33208477 10.1128/JCM.02020-20 PMC8111122

[6] Charlton CL 2020. Evaluation of six commercial mid- to high-volume antibody and six point-of-care lateral flow assays for detection of SARS-CoV-2 antibodies. J Clin Microbiol 58. 10.1128/JCM.01361-20. PMC7512179 32665420

[7] Furuya AKM 2021. Performance evaluation of antibody-based point-of-care devices intended for the identification of immune responses to SARS-CoV-2. Diagn Microbiol Infect Dis 99: 115298. 33418405 10.1016/j.diagmicrobio.2020.115298 PMC7758720

[8] Biomedomics , n.d. Documents. Available at: https://www.biomedomics.com/documents/?sub-field=infectious-disease. Accessed May 20, 2021.

[9] Centers for Disease Control and Prevntion , 2021. *Independent Evaluation of SARS-CoV-2 Antibody Test Performance.* Available at: https://www.cdc.gov/coronavirus/2019-ncov/covid-data/serology-surveillance/serology-test-evaluation.html. Accessed March 11, 2021.

[10] Cerami C 2021. High household transmission of SARS-CoV-2 in the United States: living density, viral load, and disproportionate impact on communities of color. *medRxiv*. 10.1093/cid/ciab701 PMC8436395 34383889

[11] Premkumar L 2020. The receptor binding domain of the viral spike protein is an immunodominant and highly specific target of antibodies in SARS-CoV-2 patients. Sci Immunol 5. 10.1126/sciimmunol.abc8413 PMC7292505 32527802

[12] Muller MS Bhattarai Chhetri S Basham C Rapp T Lin F-C Lin K Westreich D Cerami C Juliano JJ Lin JT, 2021. Practical strategies for SARS-CoV-2 RT-PCR testing in resource-constrained settings. *medRxiv*. 10.1101/2021.03.10.21253173. PMC8230941 34280773

[13] Wajnberg A 2020. Robust neutralizing antibodies to SARS-CoV-2 infection persist for months. Science 370: 1227–1230. 33115920 10.1126/science.abd7728 PMC7810037

[14] Greaney AJ Loes AN Crawford KHD Starr TN Malone KD Chu HY Bloom JD, 2021. Comprehensive mapping of mutations in the SARS-CoV-2 receptor-binding domain that affect recognition by polyclonal human plasma antibodies. Cell Host Microbe 29: 463–476.e6. 33592168 10.1016/j.chom.2021.02.003 PMC7869748

[15] Piccoli L 2020. Mapping neutralizing and immunodominant sites on the SARS-CoV-2 spike receptor-binding domain by structure-guided high-resolution serology. Cell 183: 1024–1042.e21. 32991844 10.1016/j.cell.2020.09.037 PMC7494283

[16] Gao Q 2020. Development of an inactivated vaccine candidate for SARS-CoV-2. Science 369: 77–81. 32376603 10.1126/science.abc1932 PMC7202686

[17] Li Z 2020. Development and clinical application of a rapid IgM-IgG combined antibody test for SARS-CoV-2 infection diagnosis. J Med Virol 92: 1518–1524. 32104917 10.1002/jmv.25727 PMC7228300

[18] Naranbhai V 2020. High seroprevalence of Anti-SARS-CoV-2 antibodies in Chelsea, Massachusetts. J Infect Dis 222: 1955–1959. 32906151 10.1093/infdis/jiaa579 PMC7499676

[19] Long Q-X 2020. Clinical and immunological assessment of asymptomatic SARS-CoV-2 infections. Nat Med 26: 1200–1204. 32555424 10.1038/s41591-020-0965-6

