# Supplementary material for: Assessment of the Field Utility of a Rapid Point-of-Care Test for SARS-CoV-2 Antibodies in a Household Cohort
Source: Am J Trop Med Hyg. 2021 Nov 24;106(1):156–9. doi: 10.4269/ajtmh.21-0592 (PMC8733539; doi:10.4269/ajtmh.21-0592)
Supplement: Supplementary file 1 [file tpmd210592.SD1.pdf]

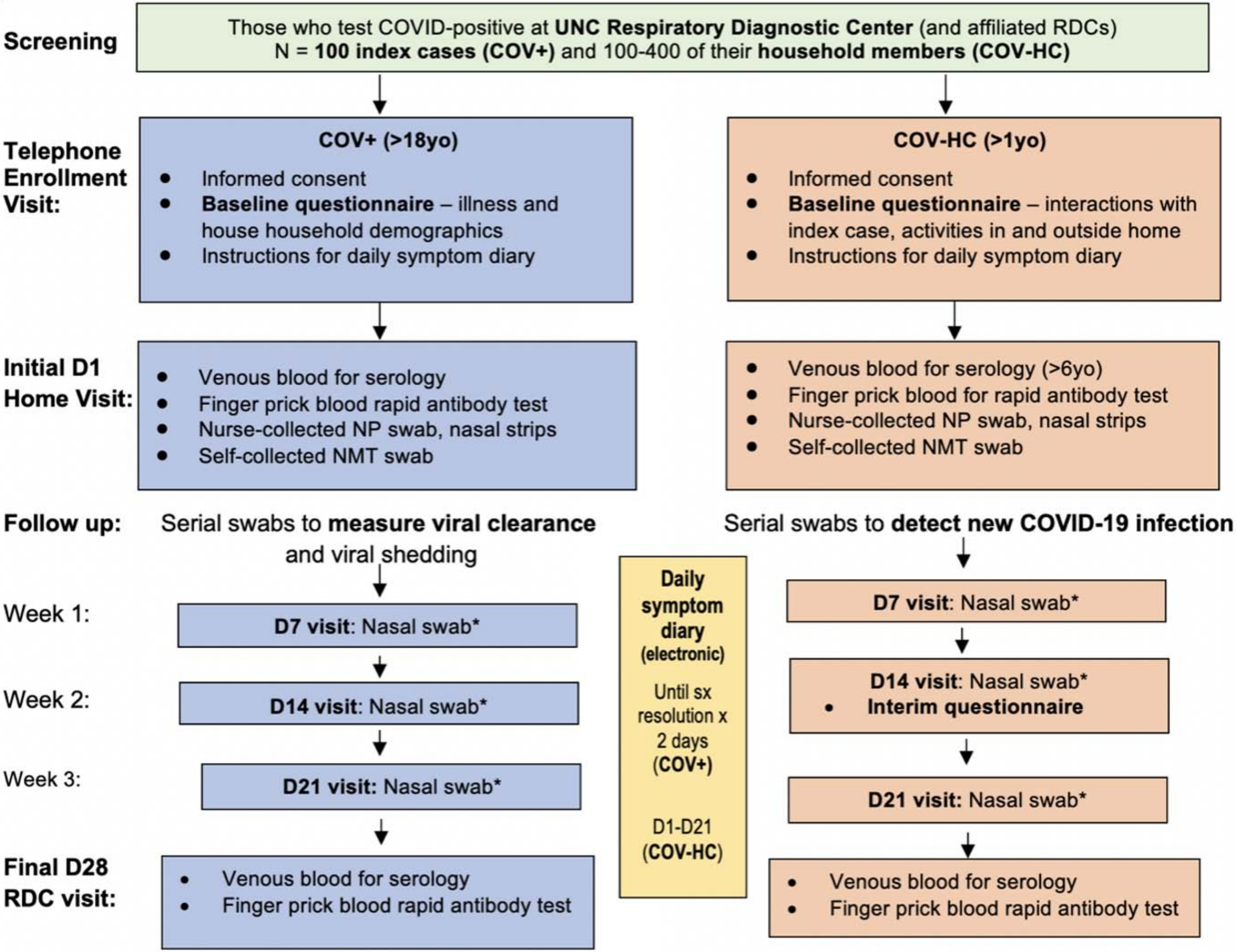

Figure S1. Schematic of CO-HOST study design.

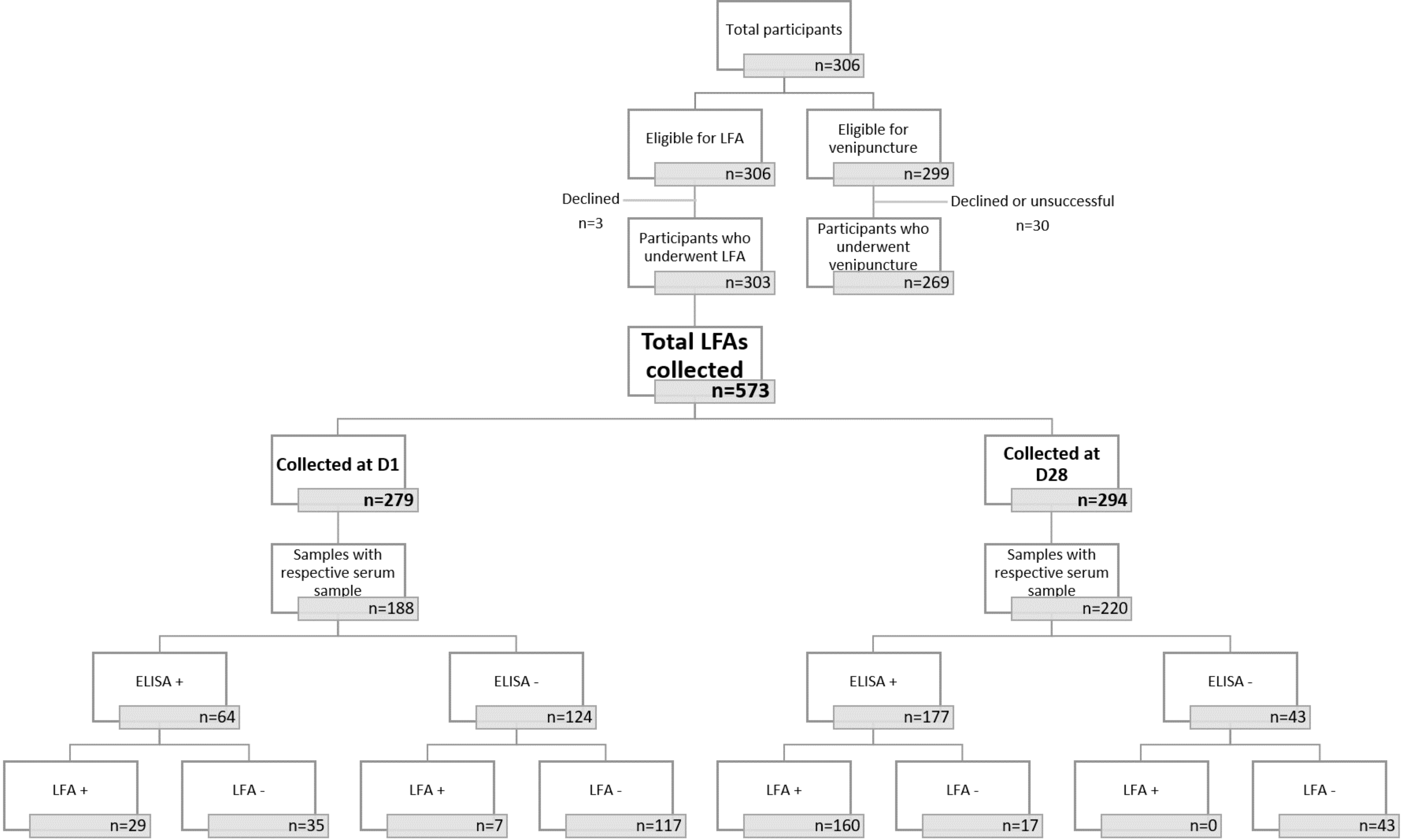

Figure S2. Number of participants and samples for LFA and venipuncture serum samples.

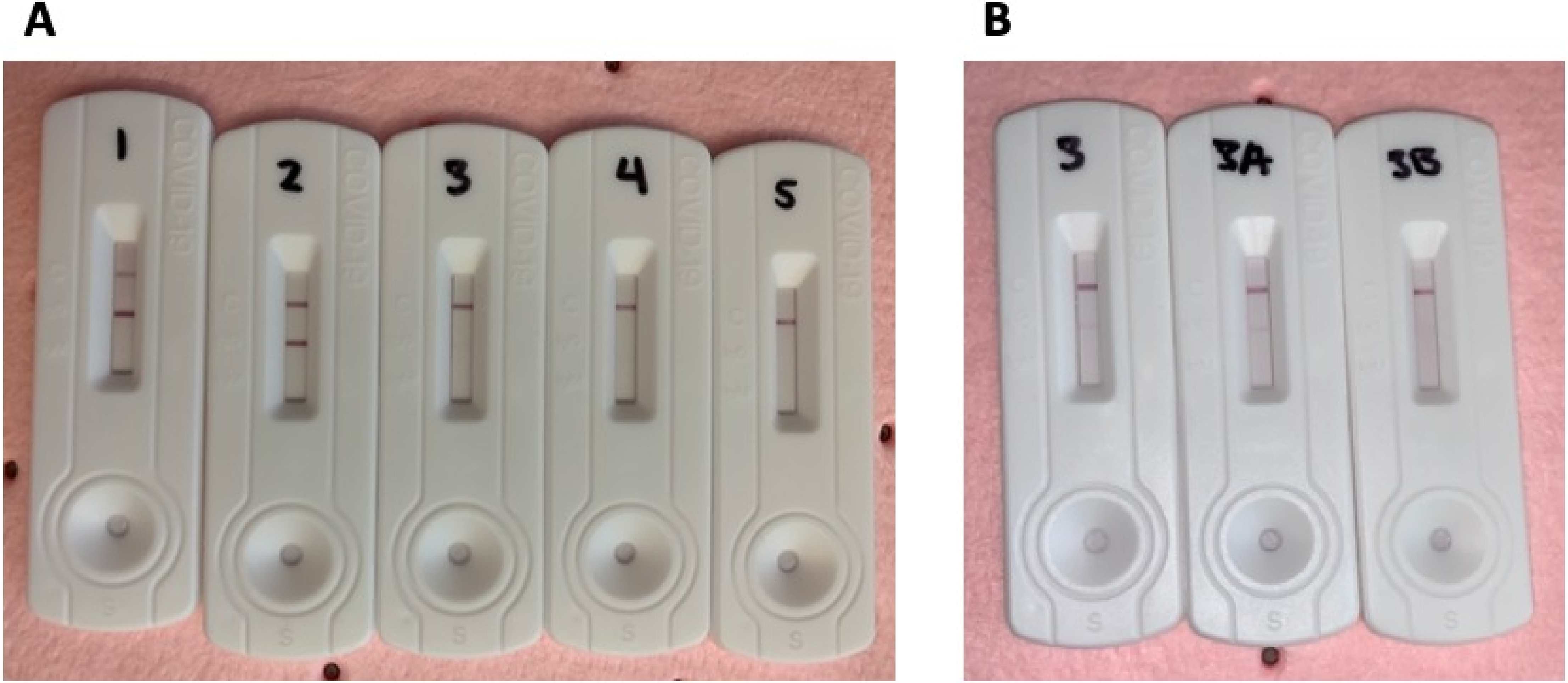

Figure S3. BioMedomics LFA performed on IgG antibody with the following concentrations for (A): (1) 1 µg (2) 100 ng (3) 10 ng (4) 1 ng (5) 100 pg in 20 µL of serum, and (B): (3) 10 ng (3A) 20 ng (3B) 30 ng in 20 µL of serum.

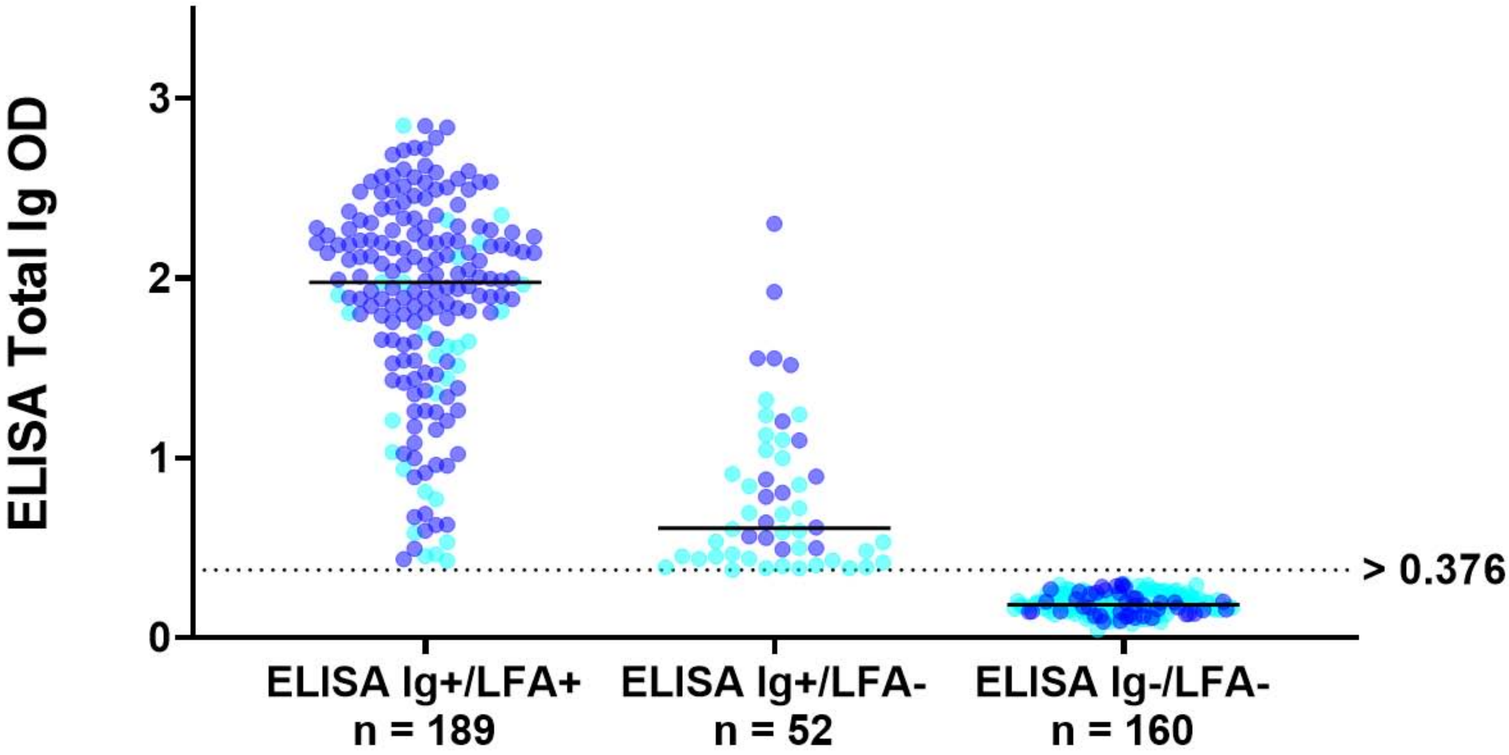

Figure S4. ELISA total Ig OD values for samples that were ELISA Ig+/LFA+ (median: 2.0), ELISA Ig+/LFA- (median: 0.6), and ELISA Ig-/LFA- (median: 0.2). Samples that were LFA-negative displayed a lower ELISA total Ig OD compared to samples that were both ELISA and LFA-positive ($p>0.0001$). The threshold of positivity was 0.376 OD. Samples tested at D1 are depicted in light blue, whereas samples tested at D28 are depicted in dark blue. Of the 52 individuals who were ELISA Ig+/LFA-, 7 had asymptomatic infection.
